# Impact of Medical Student Participation in Student-Run Clinics on Education, Residency Selection, and Patient Care: A Review of Selected Articles

**DOI:** 10.7759/cureus.26183

**Published:** 2022-06-21

**Authors:** Edwin McCray, William R Atkinson, Chelsea E McCray, Zachary Hubler, Yanal Maher, Romaric Waguia, Molly Kearney, Victoria Kaprielian

**Affiliations:** 1 Internal Medicine, Campbell University School of Osteopathic Medicine, Buies Creek, USA; 2 Internal Medicine, Campbell University School of Osteopathic Medicine, Lillington, USA; 3 Orthopedic Surgery, Campbell University School of Osteopathic Medicine, Lillington, USA; 4 Emergency Medicine, Campbell University School of Osteopathic Medicine, Lillington, USA; 5 Family Medicine, Campbell University School of Osteopathic Medicine, Lillington, USA

**Keywords:** student-run free clinic, volunteer clinic, residency selection, medical school education, student-run clinic

## Abstract

Student-run clinics (SRCs) are becoming increasingly popular at medical schools in the United States. These clinics have provided a variety of benefits, including serving disadvantaged populations and providing early clinical exposure for students. There has been no consensus on the impact of SRCs on medical education, specialty selection, and patient care. This review provides a thorough overview of student and patient outcomes as a function of medical students volunteering at SRCs. We queried PubMed for original literature published in English between the years 2000 and 2020. Inclusion criteria included primary research articles evaluating the impact of medical student participation in SRCs on education, specialty selection, and patient care. All articles included in the final review were agreed upon by three reviewers, and the pertinent data were extracted. Of 10,200 initial search results, seven papers were included in this review. These included two studies evaluating medical education, five studies evaluating residency selection, and three studies analyzing patient care. Three studies were included in multiple evaluations. The relationship between volunteering at SRCs and academic performance is unclear. Clinic volunteers had increased retention of empathy compared to non-volunteers. Additionally, clinic volunteers provided satisfactory care as determined by patient-reported outcomes, and were not more likely to pursue primary care specialties. As SRCs are increasing in number, research into the impact on medical students and patients is necessary to understand how these clinics may affect the field of health care. It is important to further evaluate how medical student involvement in SRCs can further improve patient care and outcomes.

## Introduction and background

The implementation of student-run clinics (SRCs) is a growing trend in medical education. Between 2005 and 2014, the number of SRCs doubled among US medical schools, and as of 2014, more than 75% of US medical schools have an SRC [1]. The primary function of SRCs is to provide accessible and affordable care to disadvantaged populations. In addition, medical students often participate in SRCs to improve their professional proficiency [1]. The role of medical student participation in SRCs is to provide direct patient care in a physician-supervised environment.

Participation in SRCs is considered extracurricular and students do so on a volunteer basis [2]. Given the depth and complexity of medical education, supplemental experience may augment the learning experience [3-6]. Studies have shown that early patient exposure and additional clinical experiences can improve student clinical competence [7,8]. Additionally, early exposure to clinical medicine has been shown to influence the specialty of choice among students applying for residency [9,10].

Studies on SRCs have analyzed the quality of patient care, including patient satisfaction and clinical outcomes [11-14]. Similarly, prior studies have investigated how effectively SRCs address access to care, cost of care, and other socioeconomic barriers to quality health care [15-20]. Smith et al. found that clinical outcomes for diabetic patients treated at the University of California San Diego Student-Run Free Clinic met or exceeded standards compared to other studies [12]. Also, Niescierenko et al. described students at the University of Buffalo Student-Run Free Clinic assisting uninsured individuals in applying for increased coverage, effectively helping decrease socioeconomic disparities [20]. Such studies have been largely focused on American medical schools; however, the impact may be applicable on a global scale.

In this study, we reviewed selected primary studies investigating the impact of medical student participation at SRCs on medical student education and residency selection. The effects of a variety of factors on patient care, medical education, and residency selection have been documented; however, these have not been comprehensively studied in the SRC volunteer population. The goal of this study is to understand how participation in SRCs influences the professional development of medical students in hopes of optimizing the students’ clinical experiences. Furthermore, we hope to elucidate benefits for patients and students, alike, as SRCs continue to be an integral part of health care [1].

## Review

Methods

A literature search was conducted using PubMed, along with a secondary review of the references of included articles. The search was for studies to determine the impact of medical SRCs on education, patient care, and residency selection. Search terms, as well as inclusion and exclusion criteria, are described in Table 1. The parameters included studies published from January 1, 2000, to December 1, 2020, and outcomes included clinical grades, shelf exam scores, comfort with patient interaction, empathy, confidence, and influence on specialty choice. All studies eligible for inclusion were determined by two reviewers (McCray and Kearney). Data were extracted and analyzed from the series of studies to answer the inquiry questions presented in the objectives. A third reviewer (Waguia) confirmed article inclusion. During this process, there were no disagreements in the quality or inclusion selections among the reviewers.

**Table 1:** Study selection criteria SRC: student-run clinic.

Study type	Key search terms	Inclusion	Exclusion
Education	Grades, student clinic, student-run clinic, medical student clinic, clinical grades, student clinic, exams, shelf exams, outcomes	Publication date: January 1, 2000-December 1, 2020. Language: English or with complete English translation. Studies investigating the impact of SRCs on student education. Fully published, peer-reviewed, prospective, and retrospective studies	Animal studies; study designs including abstracts, meta-analyses, poster presentations, oral presentations, systematic reviews, case reports, and book chapters; and studies including undergraduates or other students not enrolled in medical school
Residency selection	Residency, student clinic, student-run clinic, medical student clinic, residency match	Publication date: January 1, 2000-December 1, 2020. Language: English or with complete English translation. Studies evaluating SRC participation and residency selection. Fully published, peer-reviewed, prospective, and retrospective studies	
Patient care	Bedside manner, student clinic, student-run clinic, medical student clinic, confidence, comfort, empathy	Publication date: January 1, 2000-December 1, 2020. Language: English or with complete English translation. Studies evaluating SRC participation and medical student care competency. Fully published, peer-reviewed, prospective, and retrospective studies	

Data Extraction

The following data from the included studies were extracted: sample size, school location, medical school type (allopathic versus osteopathic), the time frame of data collection, and medical specialties represented at the clinic. Data on student volunteers included were grade point average (GPA), Step 1 and Step 2 Clinical Knowledge (CK) scores, specialty choice, study participant’s medical school year, empathy scores, and patient-reported outcomes.

**Figure 1:**
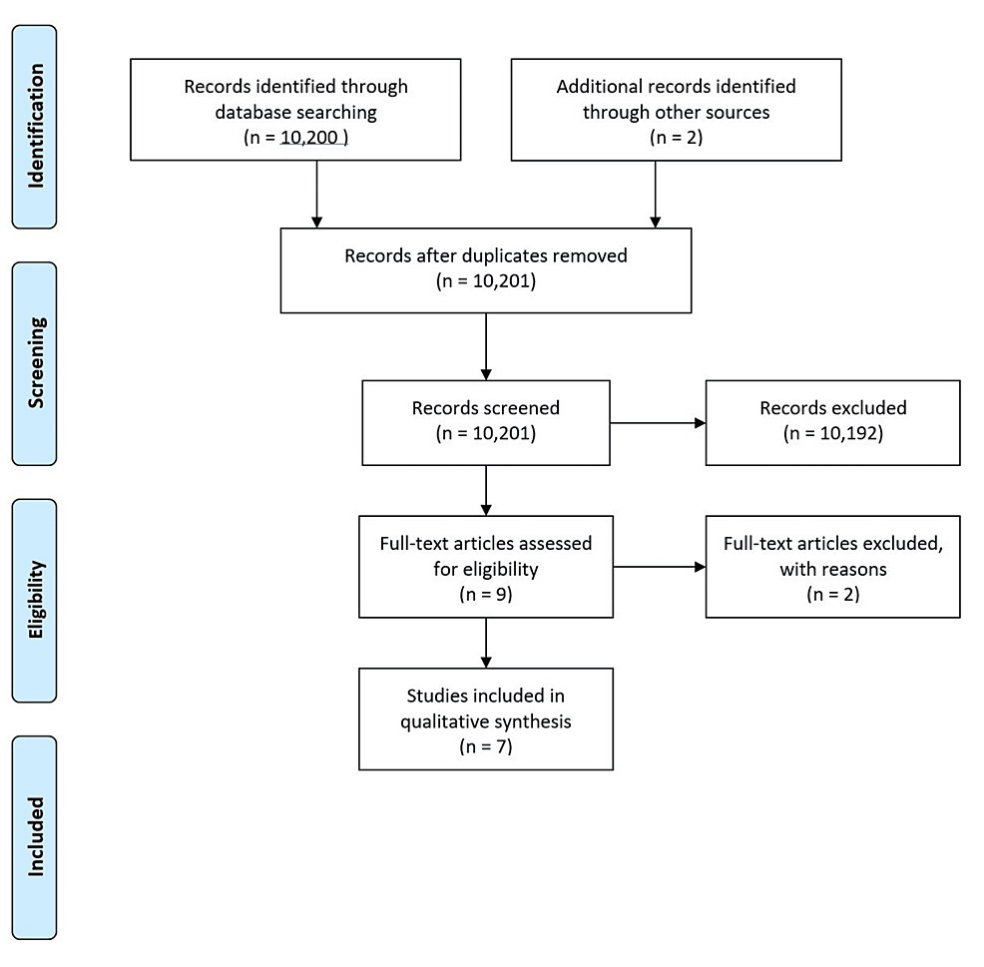
Article selection diagram

The literature search was organized into three parts based on types of outcomes from SRCs: education, residency selection, and patient care. Specific search terms for each outcome group are displayed in Table 1. Of 10,200 studies identified, 10,081 were excluded based on title and abstract alone, leaving 119 studies (Figure 1). Education, residency match, and patient outcome queries yielded 62, 31, and 26 focused results, respectively. The excluded studies did not meet our inclusion criteria if they did not contain primary data, did not include data on variables relevant to this study, or included professional students outside of medical students. Ultimately, seven studies (two retrospective studies and five prospective surveys) were included in this review, all of which are indexed by the outcome group in Tables 2-4 [21-27]. The studies conducted by Tran et al., Vaikunth et al., and Heller et al. each met the inclusion criteria of two outcome groups and are included twice each in Tables 2-4 [21,23,24].

**Table 2:** Outcomes on medical student education for students who volunteered in student-run clinics SRC: student-run clinics; GPA: grade point average; USMLE: United States Medical Licensing Examination.

Authors	Year	Study design	Outcomes
Vaikunth et al. [21]	2014	Retrospective study, 689 medical students	SRC volunteers had a higher GPA (3.59 ± 0.33 vs. 3.40 ± 0.39, p < 0.001), USMLE Step 1 score (229 ± 19 vs. 220 ± 21, p < 0.001), and USMLE Step 2 score (240 ± 18 vs. 230 ± 21, p < 0.001) compared to non-volunteers.
Stoddard et al. [22]	2011	Retrospective study, 908 medical students	There was no correlation between SRC board member status and MS1 or MS234 GPA (p = 0.064).

**Table 3:** Outcomes on residency selection for students who volunteered in student-run clinics SRC: student-run clinic.

Authors	Year	Study design	Outcomes
Tran et al. [23]	2017	Survey, 78 non-volunteers, 50 volunteers	No correlation between volunteering at the SRC and interest in becoming a primary care physician. Of the volunteers, 88% agreed or strongly agreed that their experience positively influenced their attitude toward working with underserved patients.
Heller et al. [24]	2019	Survey, 52 medical students	Surveys before and after volunteering at the SRC showed that 13/52 students had an increased desire to pursue primary care.
Brown et al. [25]	2017	Survey, 135 students	The desire to practice primary care was not influenced by the decision to volunteer at the SRC. No correlation between the number of hours volunteered and residency selection.
Tong et al. [26]	2012	Retrospective review, 115 allopathic medical schools	No correlation between a student-run clinic and the proportion of graduates practicing primary care (p = 0.286). The correlation coefficient between the presence of a student-run clinic and graduate primary care practice was -0.122.
Vaikunth et al. [21]	2014	Retrospective review, 389 medical students	Students who volunteered at the SRC were not more likely to pursue a primary care specialty (p = 0.82).

**Table 4:** Outcomes on patient care for students who volunteered in student-run clinics

Authors	Year	Study Design	Outcomes
Heller et al. [24]	2019	Survey, 34 patients	Of patients, 88% agreed or strongly agreed that they were satisfied with their care.
Modi et al. [27]	2016	Prospective survey, 103 non-volunteers, 85 volunteers	Mean empathy scores declined by 2.2 points in student-run clinic volunteers (p = 0.07). Mean empathy scores declined by 3.1 points in non-volunteers (p = 0.009).
Tran et al. [23]	2017	Survey, 78 non-volunteers, 50 volunteers	No significant difference in self-reported comfort or capability between volunteers and non-volunteers.

Results

Two of the seven studies included in the review examined medical student educational outcomes. Stoddard et al. conducted a retrospective review analyzing the impact of SRC participation on medical student grades at the University of Nebraska Medical Center College of Medicine (UNCOM) [22]. UNCOM matriculants from 1999 to 2006 were split into two groups: SHARING Clinic (an SRC) student board members and non-board members. Student board members averaged between two and 10 hours per week volunteering in the SRC in comparison to non-board members, who rarely volunteered more than one time per semester. Stoddard et al. collected and compared the first, second, third, and fourth-semester GPA of 87 SHARING Clinic student board members and 821 non-board members [22]. Despite more frequent participation at the SRC by SHARING Clinic student board members, the study found no significant difference in grades between the two groups [22].

Vaikunth et al. examined Clinica Esperanza's (an SRC) participation at the University of Tennessee Health Science Center (UTHSC) with no minimum GPA parameters [21]. The study included medical students at UTHSC who graduated between 2008 and 2012 and stratified students based on SRC volunteering. Comparison groups consisted of non-volunteers vs. any amount of volunteering, volunteering less than 7.5 hours vs. volunteering more than 7.5 hours, and volunteering less than 15 hours vs. volunteering more than 15 hours. Compared to the 548 non-volunteers, 141 students with any SRC volunteering had significantly higher cumulative GPAs, United States Medical Licensing Examination (USMLE) Step 1 scores, and USMLE Step 2 CK scores. However, there was no significant correlation between the number of hours volunteered and academic achievement within the volunteer group.

In addition to educational outcomes, Vaikunth et al. examined the association between SRC participation and UTHSC student residency selection, specifically primary care [21]. The study defined primary care selection as matching "categorical programs in medicine, pediatrics, combined medicine/pediatrics, obstetrics/gynecology, and family practice." Using the same study sample outlined above, Vaikunth et al. concluded that there was no significant difference in primary care residency selection [21]. All volunteer groups and non-volunteers entered primary care residencies at a similar rate.

At the University of Central Florida College of Medicine (UCF COM), 128 first- and second-year medical students completed a survey assessing their “knowledge/skill/self-efficacy/comfort level/interest/attitude regarding the underserved” [23]. Between the two groups, KNIGHTS Clinic volunteers vs non-volunteers, 62% of volunteers strongly agreed that the clinic has a positive influence on their attitude toward working with the underserved. However, there was no statistical significance when comparing mean responses on the survey between non-volunteers and volunteers. Furthermore, there was no significant difference between the groups’ self-reported comfort and capability while treating patients.

Another study conducted at the KNIGHTS Clinic in 2017 looked at the effect volunteering had on entering into primary care, as well as the influence gender had on decisions to volunteer at the clinic [25]. The researchers sent out a survey to first-, second-, and third-year medical students at UCF COM. Data were collected between November and December 2014 from 135 students; 79 were volunteers and 56 were not. The data did not show a statistically significant difference between volunteers and non-volunteers in their choice to enter primary care. They also found that the volunteers’ gender did not impact the extent of volunteering or their desired specialty.

An observational study done at the University of Cape Town in South Africa evaluated patient satisfaction and how volunteering at the Students' Health and Welfare Centres Organisation (SHAWCO) affected their desire to go into primary care [24]. The researchers observed medical examinations between 52 medical students and 34 patients between May 25 and July 15, 2018. This study showed that volunteering in the clinic did not change the students’ desire to pursue primary care. Patient satisfaction was found to be very high with 88% of patients stating they strongly agreed to being satisfied with their care [24]. It was also discovered that the patients rated the medical students higher on listening skills than the attending physicians.

A 2017 study that was published evaluated the empathy changes for the class of 2015 at Sidney Kimmel Medical College in Philadelphia [27]. At the beginning of medical school and at the end of their third year, the students completed a Jefferson Scale of Empathy (JSE) questionnaire. For students who volunteered at the JeffHOPE clinic at least once during their first two years (45% of the sample population), there was not a statistically significant decline in empathy scores. The non-volunteer group did show a statistically significant (p = 0.009) decline in empathy levels.

Rather than examining residency selection outcomes at an individual medical school, Tong et al. investigated the connection between having an SRC at an allopathic medical school and the number of students that go into primary care [26]. In 2005, Tong et al. used the American Medical Association Physician Masterfile to identify currently practicing primary care physicians from 72 medical schools that had clinics, and 43 schools that did not. The researchers did not find an association between schools with SRCs and a future in primary care.

Discussion

Previous studies have shown that early clinical exposure, such as prior clinical employment, impacts student education [28], yet literature is limited in evaluating this in the setting of SRCs. Our review used the PubMed search engine (MEDLINE database) to survey recent literature to evaluate the impact of medical student participation in SRCs on academic performance, patient care, and residency selection. We found that patient care and academic performance may be positively influenced by participation in SRCs. Additionally, our study is the largest study to demonstrate a lack of evidence that SRC participation impacts the pursuit of primary care residencies.

Medical Education and Academics

One objective of our study was to assess the effect of SRC involvement on student education outcomes. There is significant evidence that demonstrates an academic advantage for students with early clinical experience [5-7,28]. Specifically, research has shown a correlation between pre-matriculation clinical experience [29] and higher clinical volumes [30] with increased grades and board scores. However, we found conflicting evidence on the impact of SRC participation on academic performance. In a single-institution study conducted at the University of Nebraska Medical Center College of Medicine, Stoddard et al. found no difference in GPA based on the extent of SRC involvement (2-10 hours per week vs. once per semester) [22]. Conversely, Vaikunth et al. reported that those involved in SRCs had a higher GPA, and higher Step 1 and Step 2 CK scores [21]. Prior published literature support both conclusions [29,31], and the reasons for the discrepancies are not entirely clear. One potential hypothesis is that students who are interested in highly competitive specialties such as neurological surgery, plastic surgery, dermatology, otolaryngology, radiation oncology, orthopedic surgery, ophthalmology, and urology [32] may be more likely to engage in extracurricular activities. According to the National Resident Matching Program (NRMP) Charting Outcomes, a biennial descriptive report highlighting the characteristics of students’ performance in the NRMP match, students who successfully match into highly competitive specialties tend to engage in more extracurricular activities and demonstrate higher academic performance [33,34]. This could explain the correlation between SCR participation and GPA and board scores found in Vaikunth et al.'s study [21], as the relationship may be impacted by confounding factors like the specialty of pursuit. Likewise, the lack of difference in GPA between SRC board members and non-board members in Stoddard et al.'s study [22] may be similarly affected by confounding factors. The inability to distinguish those who are more externally driven by factors such as the competitiveness of residency specialty of choice makes it difficult to truly elucidate the relationship or lack of relationship between SRC participation and GPA. Alternatively, the variation in outcomes amongst studies may be the result of school or student-specific factors [35,36], which are not accounted for in either of the included studies.

Residency Selection

Currently, there is a considerable shortage of physicians in the US, which is relatively higher in the primary care specialties [37]. As such, we sought to evaluate the relationship between SRC volunteers and the pursuit of residency in a primary care specialty. In the literature, there is heterogeneity in the working definition of medical specialties of primary care; however, many consider the primary care specialties to include family medicine, general internal medicine, general pediatrics, and obstetrics and gynecology [38]. Out of the five studies addressing residency selection outcomes as a function of volunteering at SRCs, four studies found no significant relationship [21,23,25,26]. Of note, the remaining study demonstrated that 13/52 (25%) of the study participants reported an increased desire to pursue primary care specialties after volunteering at SRCs [24]. Previously published literature shows that residency specialty selection is multifactorial [39]. However, these data are significantly limited. Thus, the ability to compare our findings with the general consensus is also limited. The overall trend in the literature we reviewed demonstrates that a relationship between SRC participation, including primary care specialties, may not exist. This may be the result of a lack of data granularity in the included studies; factors such as mentorship experiences, perceived personal ability, rigor and length of training, and academic performance may have a heavier impact on specialty choice [10,33]. Additionally, the educational structure and environment unique to each college of medicine may shape students’ specialty of choice decisions. Cummings found that students attending osteopathic colleges of medicine may be influenced to pursue primary care specialties due to relatively increased difficulty in matching non-primary care specialties as compared to their allopathic counterparts [40]. Although neither of the included studies was conducted at osteopathic medical schools, the principle of perceived disadvantages may apply disproportionally based on school prestige, mission, specialty options of home programs, or other school-specific factors.

Patient Care

The patient-provider interaction is at the center of the healthcare system, and even more so in the new era of patient-centered practice [41]; thus, patient satisfaction is an important measure. Previous literature demonstrates positive outcomes in patients seeking care at SRCs [11,13,15,20]. Additionally, studies show that both patient satisfaction and clinician empathy are associated with better clinical outcomes [42-44]. We found three studies that evaluated the impact of SRC participation on patient care, two of which showed positive outcomes as measured by patient satisfaction scores and evidence of increased empathy in student volunteers [24,27]. Our findings may be explained, in part, by the disadvantaged patient population generally treated at SRCs. Patients with lower socioeconomic status are more likely to be uninsured or underinsured [45,46]; therefore, they are likely to have less frequent encounters with healthcare providers. As such, patient-reported satisfaction scores may be biased by lack of experience for comparison. Alternatively, the reported satisfaction scores may be influenced by gratitude for free or discounted services or other indirect patient-care factors. A third study found no difference in student-reported confidence or comfort in treating patients between volunteers and non-volunteers [23]. Since the perception of personal capability may influence the patient-provider interaction, medical student confidence and comfort may serve as a proxy measure of the level of care delivered.

Limitations

As with all secondary research, our findings are limited by the search tools utilized and the articles included. While each of the review studies was assessed for feasible methodology and relevant objectives, most contained lower-quality methods; this includes low sample sizes, the use of survey methodology, and no multi-institution studies. Additionally, variables such as education level prior to medical school, work experience, medical college admissions test scores, and personal demographic characteristics were not accounted for. We attempted to mitigate these limitations by aiming to assess general objectives and refraining from implying causation. The lack of granularity in data contained in the included studies only allowed us to draw associations and highlight potential trends. It will be beneficial to conduct more granular, multi-center primary studies, reviews, and meta-analyses to validate our findings. Additionally, a bias toward US-specific medical school performance measures and residency selection processes limited this study.

## Conclusions

The increasing practice of medical student volunteering in SRCs has led to research regarding the impact of this type of early clinical exposure on patient and student outcomes. We report several potential academic and personal development advantages associated with SRC participation, including increased student GPA, USMLE Step 1 scores, and USMLE Step 2 scores. However, we found no evidence of a correlation between SRC volunteering and residency specialty of choice. Given the possible benefits and relatively low negative effects in SRC utilization, it is important to assess how incorporating these experiences into medical school training can be refined to further improve outcomes.
